# Awareness and Attitude towards Human Papillomavirus (HPV) Vaccine among Medical Students in a Premier Medical School in India

**DOI:** 10.1371/journal.pone.0040619

**Published:** 2012-07-31

**Authors:** Deeksha Pandey, Vidhi Vanya, Saurav Bhagat, Binu VS, Jyothi Shetty

**Affiliations:** 1 Department of Obstetrics and Gynecology, Kasturba Medical College, Manipal University, Manipal, Karnataka, India; 2 MBBS Student, Kasturba Medical College, Manipal University, Manipal, Karnataka, India; 3 Department of Statistics, Manipal University, Manipal, Karnataka, India; The University of Hong Kong, Hong Kong

## Abstract

**Background:**

As preventing cancer with the help of a vaccine is a comparatively new concept, awareness and education about it will have important implication in the implementation of this strategy.

**Materials and Methods:**

Present explorative questionnaire based survey included 618 MBBS students for final analysis.

**Results:**

Majority of participants (89.6%) were well aware of the preventable nature of cervical cancer. Most of them (89.2%) knew that necessary factor responsible for cervical cancer is infection with high risk HPV. Awareness regarding the availability of vaccine against cervical cancer was 75.6%. Females had a better awareness regarding availability of vaccine, target population for vaccination and about the catch up program. Overall acceptance of HPV vaccine among the population studied was 67.8%. Medical teaching had a definitive impact on the understanding of this important public health issue. Females seemed to be more ready to accept the vaccine and recommend it to others. For our study population the most common source of information was medical school teaching. Majority of participants agreed that the most important obstacle in implementation of HPV vaccination program in our country is inadequate information and 86.2% wanted to be educated by experts in this regard.

**Conclusion:**

HPV vaccine for primary prevention of cervical cancer is a relatively new concept. Health professional will be able to play a pivotal role in popularizing this strategy.

## Introduction

Cervical cancer is the second most common cancer among women worldwide. About 500,000 women are diagnosed with cervical cancer contributing to around 270,000 deaths, across the globe every year. Out of these, the burden of 230,000 (85%) deaths is owned by developing countries, with bare minimal resources to cope with the situation [1]. In India alone there are an estimated 132,000 new cases and 74,000 deaths each year [2]. The discovery that human papillomavirus (HPV) is responsible for virtually all cervical cancers [3], [4], [5] opens exciting new possibilities for controlling this disease.

As preventing cancer with the help of a vaccine is a comparatively new concept, awareness and education will have important implication in the implementation of this strategy. It should be well understood that the mere availability of an effective vaccine is not synonymous with an effective vaccination program.

We hypothesized that awareness programmes conducted at various levels addressing tailored issues will help to successfully implement HPV vaccination in our country. We chose medical students (age group: 17–25) for the simple reason that in a few years these students will be the practicing clinicians, and will be sought by the population as the first line information resources and can play a pivotal role in spreading awareness among a wide range of population. Educational initiatives targeting health care professionals have a definitive role in fostering vaccine acceptance. As demonstrated by Gonik et al educational interventions can positively influence immunization-related practice patterns [6]. Results obtained from present study may also be useful at the policy level to implement awareness programs among the health care professionals about this important public health issue.

## Results

A total of 641 students participated in the study. Twenty three questionnaires were incomplete and were excluded from the final analysis. Out of the 618 participants included in the final analysis, 268 (43.4%) were males and 350 (56.6%) were females. Most of them 49.4% were in the age group of 20–22 years, 36.4% belonged to 17 – 19 years, while 14.2% were 23 –25 years old. Two seventy seven (44.8%) students were in the final year of medical school (test group). Three forty one (55.2%) had recently joined medical school, were studying preclinical subjects with bare minimum exposure to patients and clinical teaching. This group served as control group for our study to evaluate the contribution of medical education, in practical issues like cervical cancer prevention. Detailed characteristics of the participants are presented in table 1.

**Table 1:** Demographic characteristics of the population studied.

Participant characteristics	Number of Participants (n = 618)	Percent Distribution (%)
1. Sex		
Male	268	43.4
Female	350	56.6
2. Education level		
Non clinical	341	55.2
Clinical	277	44.8
3. Age		
17–19	225	36.4
20–22	305	49.4
23 & above	88	14.2

### 1. Awareness

#### 1.1. Awareness regarding preventable nature of cervical cancer

Five hundred and twenty four (84.8%) participants were well aware of the preventable nature of cervical cancer (Table 2). Two hundred ninety nine (85.4%) females as compared to 225 (84%) males knew that cervical cancer can be prevented (Table 3). The difference in the awareness regarding the preventable nature of cervical cancer was significant (p = 0.012) among test group (246, i.e. 88.8%) as compared to the control group (278, i.e. 81.5%) (Table 4).

**Table 2:** Awareness about cervical cancer prevention through HPV vaccination.

Clubs	Awareness among participants	
	frequency	%
Awareness regarding		
1. preventable nature of cervical cancer	524	84.8
2 etiology of cervical cancer	551	89.2
3. availability of vaccine	467	75.6
4. target population for vaccination	426	68.9
5. need to vaccinate men	156	25.2
6. catch up program	509	82.4
7. vaccine dosage	249	40.3
8. protective efficacy	472	76.4

**Table 3:** Comparison of awareness among males & females.

Clubs	Males Aware (n = 268)		Females Aware (n = 350)		P value
	frequency	%	frequency	%	
Awareness regarding					
1. preventable nature of cervical cancer	225	84	299	85.4	0.613
2. etiology of cervical cancer	236	88.1	315	90.0	0.442
3. availability of vaccine	176	65.7	291	83.1	<0.001*
4. target population for vaccination	166	61.9	260	74.3	0.001*
5 need to vaccinate men	76	28.4	80	22.9	0.119
6. catch up program	230	85.8	279	79.7	0.048*
7. vaccine dosage	104	38.8	145	41.4	0.510
8. protective efficacy	196	73.1	276	78.9	0.097

**Table 4:** Comparison of awareness according to educational level.

Clubs	Non clinical (control)		Clinical (test)		P value
	frequency	%	frequency	%	
Awareness regarding					
1. preventable nature of cervical cancer	278	81.5	246	88.8	0.012*
2. etiology of cervical cancer	282	82.7	269	97.1	<0.001*
3. availability of vaccine	245	71.8	222	80.1	0.017*
4. target population for vaccination	227	66.6	199	71.8	0.159
5. need to vaccinate men	69	20.2	87	31.4	0.005*
6. catch up program	282	82.7	227	81.9	0.808
7. vaccine dosage	128	37.5	121	43.7	0.121
8. protective efficacy	239	70.1	233	84.1	<0.001*

Three forty one (55.2%) had recently joined medical school, were studying preclinical subjects with bare minimum exposure to patients and clinical teaching. This group served as control group for our study to evaluate the contribution of medical education, in practical issues like cervical cancer prevention.

#### 1.2. Awareness regarding etiology of cervical cancer

Cervical cancer is now known to be caused by high risk HPV, overall awareness of this fact was 89.2% (Table 2). Three hundred and fifteen (90%) females and 236 (88.1%) males knew this (Table 3). Among the test group this awareness was 97.1% (269) as compared to the control group where this awareness was 82.7% (282), the difference is statistically significant (p<0.001) (Table 4).

#### 1.3. Awareness about availability of vaccine for cervical cancer

Awareness regarding the availability of vaccine against cervical cancer was 75.6% (n = 467). Eighty three percent (n = 291) females as compared to 65.7% males were aware of this. This difference was statistically significant (p<0.001) (Table 3). Comparing educational groups for this particular issue we found that the awareness regarding the availability of vaccine was more in the group who were exposed to clinics (71.8% for control versus 80.1% for test group; p = 0.017) (Table 4).

#### 1.4. Awareness regarding target population for HPV vaccination

Overall awareness regarding the target population for HPV vaccination was 68.9% (426). Two hundred and sixty females (74.3%) and 166 (61.9%) males knew this (p = 0.001) (Table 3). Once again this awareness showed no statistical difference between the educational groups (66.6% for control versus 71.8% for test group) (Table 4).

#### 1.5. Awareness regarding the need to vaccinate men

This was the lowest scoring item in the whole questionnaire with overall correct response of 25.2% only (Table 2). Only 28.4% (76) males and 22.9% (80) females chose the correct response (Table 3). Though only 31.4% (87) among test group, compared to 20.2% (69) among the control group could answer this correctly, the difference is statistically significant (p = 0.005) (Table 4).

#### 1.6. Awareness regarding the catch up program

Five hundred and nine subjects (82.4%) knew about the catch up program (Table 2), which included 230 (85.8%) males and 279 (79.7%) females. Awareness of catch up program showed no difference between the educational groups (Table 4).

#### 1.7. Awareness of vaccine schedule

Less than half of the participants knew about the correct vaccine schedule (Table 2). Only 38.8% males and 41.4% females knew that HPV vaccine requires three dosage (Table 3). Forty four percent students from test group, as compared to 37.5% among the control group had adequate information about the correct vaccination schedule (Table 4).

#### 1.8. Awareness regarding the protective efficacy

Overall awareness regarding the protective efficacy of HPV vaccine was 76.4% (Table 2). Two hundred and seventy six (73.1%) females and 196 (73.1%) males knew this (Table 3). The difference was found to be statistically significant between control and test group (84.1% versus 70.1%; p<0.001) (Table 4).

### 2. Acceptance of HPV vaccine

Overall acceptance of HPV vaccine among the population studied was 67.8%. Females seemed to be more ready to accept the vaccine and recommend it to others. Educational level of the subject did not make any bias as far as the acceptance of this concept was concerned. (Table 5).

**Table 5:** Acceptance of HPV vaccine.

	YES (%)	No (%)	DON'T KNOW (%)	P value
Overall	67.8	18.6	13.4	
Sex wise				<0.001**
males	53.0	31.3	15.7	
females	79.4	8.9	11.7	
Education wise				0.140
Non clinical (controls)	65.6	18.5	15.9	
Clinical (test)	70.8	18.8	10.5	

### 3. Sources of information

**Figure 1:**
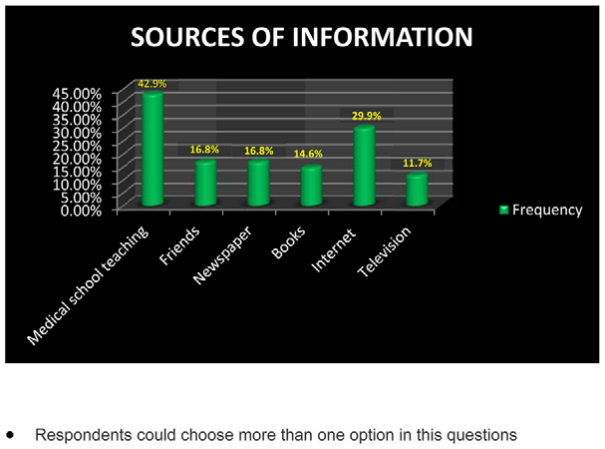
Sources of information regarding the HPV vaccination. (Respondents could choose more than one option in these questions).

Most common source of information for our study population was medical school teaching (42.9%). Other sources in order were internet (29.9%), friends (16.8%), newspaper (16.8%), books (14.6%) and television (11.7%). (Figure 1).

About 14% of our study participants had been questioned by friends or family about HPV vaccination.

### 4. Obstacles for implementation of HPV vaccination program

**Figure 2:**
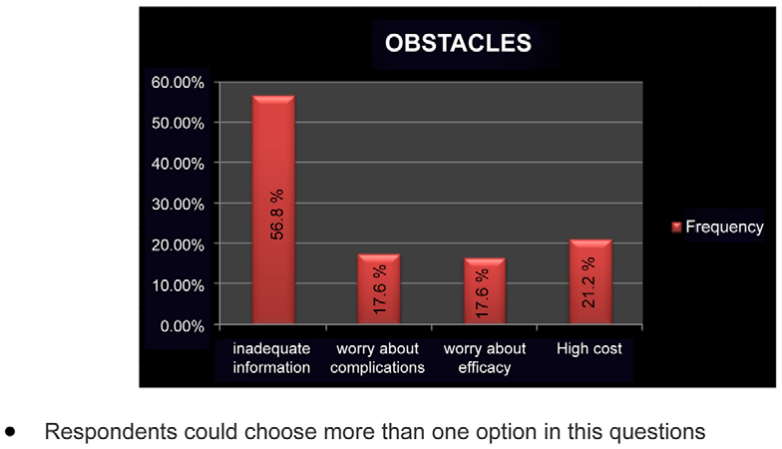
Obstacles for implementation of HPV vaccination program. (Respondents could choose more than one option in these questions).

According to the belief of our study population thought high cost (21.2%), fear of complications (17.6%) and worry about efficacy (16.7%) are the important obstacles for implementation of HPV vaccination program. However more than half (56.7%) agreed that most important problem is inadequate information (Figure 2). Earlier studies in China and Turkey too found high monetary cost, as the most important perceived barrier for implementation of HPV vaccine in their population [7], [8].

## Discussion

Majority of participants in our study were well aware of the preventable nature of cervical cancer. Most of them knew about its viral etiology. A study conducted by Saha et al in Kolkata, India revealed a very low level of awareness among the graduate and postgraduate students about this important public health issue [9]. Another recently published questionnaire based study conducted to find out awareness about the risk factors for cervical cancer among the educated youth in India, Sri lanka and Nepal. The average awareness in this regard was found to be cervix cancer was 66% in India, 58.8% in Nepal and 57.7% in Srilanka [10]. The difference is because the population studies by us are students in medical profession, which is not the reflection of general population. Awareness regarding the availability of vaccine against cervical cancer in a study conducted among women attending routine gynecological care in Belgium was only 50% [11]; whereas in our cohort of medical students it was 75.6%. Females had a better awareness regarding availability of vaccine, target population for vaccination and about the catch up program. We found that medical teaching had a definitive impact on the understanding of this important public health issue, with regards to etiology, of cervical cancer, availability of the vaccine and its protective efficacy. However, there were comparable awareness regarding target population for vaccination and vaccine dosage among the controls. Overall acceptance of HPV vaccine among the population studied was 67.8%. Females seemed to be more ready to accept the vaccine and recommend it to others. For our study population the most common source of information was medical school teaching followed by internet, friends, newspaper, books and television respectively.

The major obstacles to implementation of HPV vaccine programs in our country as mentioned by Bhatla N et al included cost, acceptability, lack of public awareness and infrastructure, concern about unknown side-effects and social and religious barriers [12]. In their review article by Bharadwaj at el, high cost of the vaccines was stated as the major concern for mass vaccination program in India [13]. Majority of participants agreed that most important obstacle in implementation of HPV vaccination program in our country is inadequate information. Even though this subject is the part of medical curriculum 86.2% wanted to be educated by experts immediately following the questionnaire session, which shows the potential of education programs where interrogation is followed by teaching.

As per our knowledge present study is the first initiative to find out the level of awareness about one of the currently most discussed topic of cervical cancer vaccine, among the future health care providers. The other strength of our study was that we did not stop at just finding out the awareness and attitude rather in our second session we tried to educate and inform them. This kind of informative session to the receptive minds immediately following the interrogative session, in our opinion will have a positive impact. However the study had some limitations. The study was based on convenience sampling. Medical students from only one medical school were included which might not reflect the overall awareness of medical students in India. Inclusion of other health care workers likes interns, post graduates and nursing students in future studies might increase the impact. In the bigger picture all health workers need to be educated about how to help patients to understand the advantages and limitations of this newly popularized cervical cancer prevention strategy. None of the students showed their unwillingness to participate in the study in the beginning. However, as mentioned earlier a total of 23 questionnaires were incomplete and were excluded from the final analysis. Among these 18 (13 males and 5 female participants) and were from the control group 5 (all males) were from the test group, which might have causes some attrition bias in the final results. These limitations of this study should be considered before interpreting the findings.

To conclude HPV vaccine for primary prevention of cervical cancer is a relatively new concept. This concept will be amalgamated in practice only with its increased understanding by the provider and the recipient. Health professional will be able to play a pivotal role in popularizing this strategy. Our academic curriculum in the medical schools needs to focus more on such high priority practical upcoming issues. Better understanding of the major preventive public health issues by health care professionals will definitely be propagated well in the society. All medical students (today) will not be educators in medical schools, but they are the trustworthy sources to the society full of information in this internet era.

## Materials and Methods

Present explorative questionnaire based survey, followed the ethical guidelines of the Institutional Review Board (Kasturba Hospital Manipal) and attained its approval on 11^th^ of February 2010 (IEC 011/2010).

All students of both sexes studying MBBS were included in the study. The only criterion for exclusion was unwillingness of a student to participate in the study. Students in batches of around 100 were introduced to the study by a consultant Gynecologist and were encouraged to participate in the study. Students were allowed to ask questions regarding their participation, were provided with a written summary of information about the study. The personal right to withdraw from the survey at any moment was ensured. Written consent for participation was obtained which was collected separately after it had been signed by the participant in order to avoid personal identification. Thus anonymity and confidentiality of the participants was guaranteed.

Following this, the study was conducted in two steps: step 1) Students were asked to answer the questionnaire having 20 questions to acquire information related to their baseline understanding of the disease and concept of vaccination, level of acceptance of this approach and zeal to learn more about the subject; step 2) answers were provided by the consultant Gynecologist in an effort to educate the participants to make them aware about the facts related to HPV vaccination. Later on responses to the questionnaire were collated by the investigators and were analyzed statistically. In order to decrease the bias of medical teaching, responses from participants at different educational level were analyzed separately. As cervical cancer is a primary health problem of women, awareness and acceptance among females was analysed separately and compared with that of males.

Survey instrument: Questionnaire (Questionnaire S1, Questionnaire S2) – A questionnaire having 20 questions has been prepared keeping in mind the awareness required to be disseminated among the society in relation to cervical cancer and HPV vaccination related issues. The questionnaire contains individual questions to find out acceptability of HPV vaccine among the population studied and their zeal to know more about this current issue of concern. The questionnaire is in English, which is the language of instruction for medical course in India. For construction and content validity, of the questionnaire was reviewed by two independent clinicians (Dr JS and Dr VP). There was 80% agreement on the 20 questions and their wordings. We tested the questionnaire for face validity in a pilot study on 10 interns and postgraduate students to ascertain if the questions were acceptable and their wording was well understood by the respondents. ***(Kindly refer to the supplementary file: Questionnaire S1).

### 1. Analysis

Each questionnaire contained 20 questions: 15 were related to awareness & 5 were related to acceptance of HPV vaccine. First 15 questions were further clubbed into 8 groups (we called them clubs) addressing following issues: club 1) preventable nature of cervical cancer (Q1+Q2), club 2) etiology of cervical cancer (Q3), club 3) availability of vaccine for cervical cancer prevention (Q4 + Q5), club 4) awareness regarding the target population for vaccination (Q6), club 5) need to vaccinate men (Q7), club 6) awareness regarding the catch up program (Q8 +Q9+ Q10), club 7) awareness regarding the dose (Q11), club 8) awareness regarding protection provided by HPV vaccine (Q12 + Q13+ Q14+ Q15). Responses for all questions were graded 0, 1 or 2. If the answer chosen showed that this concept of student was totally wrong or was detrimental to the society, it was given a grade of 0. If the student accepted that he/she dosen't know (as they are aware and accepting that they do not know, and hopefully will try to find out the same before advising) or it was near to the correct option, it was graded as 1. Correct response was given a grade of 2. Grades for individual questions are provided in the supplementary file ***(Kindly refer to the supplementary file: Questionnaire S2). Notably a cut off was set for each club based on required awareness for those set of questions by medical professionals.

Data obtained were analyzed using SPSS statistical software version 16. The responses of the participants to questions were analyzed according to the stratification. Chi square test was used to assess the significance of the responses and a p value <0.05 was considered statistically significant.

## Supporting Information

Questionnaire S1 **Questionnaire which was used as the survey instrument during the study.**
(DOC)

Questionnaire S2 **Questionnaire S1 with response scoring system used for analyzing the data.**
(DOC)
