# Supplementary material for: Awareness and Attitude towards Human Papillomavirus (HPV) Vaccine among Medical Students in a Premier Medical School in India
Source: PLoS One. 2012 Jul 31;7(7):e40619. doi: 10.1371/journal.pone.0040619 (PMC3409219; doi:10.1371/journal.pone.0040619)
Supplement: Questionnaire S1 — Questionnaire which was used as the survey instrument during the study. (DOC) [file pone.0040619.s001.doc]

**Awareness and acceptance of HPV vaccination among medical students**

**Study no (official use only)**

**Please answer the questions by ticking the box after your chosen response**

**Age: Sex: M F**

**Course: MBBS BDS:**

**Semester:**

1. **All cancers are preventable.**

**True False don’t know**

1. **Cervical cancer is preventable.**

**True False don’t know**

1. **Cervical cancer is caused by**

**Bacteria Virus Fungi None of these**

1. **Is there any vaccine available for cervical cancer?**

**Yes No don’t know**

1. **Is the cervical cancer vaccine available in India?**

**Yes No don’t know**

1. **Which age group HPV vaccine should be given?**

**0-10 y 10-30y 30-50y 50 & above**

1. **Can it be given to boys?**

**Yes No don’t know**

1. **Can it be given to a sexually active girl?**

**Yes No don’t know**

1. **Do girls/women need to be screened for HPV before getting vaccinated?**

**Yes No don’t know**

1. **Can it be given to a woman already having HPV infection?**

**Yes No don’t know**

1. **How many doses of HPV vaccine are required for protection?**

**One two three four**

1. **Is it safe to have multiple sexual partners after full course of HPV vaccine?**

**Yes No don’t know**

1. **Is it safe to have sex without condoms after HPV vaccine?**

**Yes No don’t know**

1. **Do girls/women who have already been vaccinated, require cervical cancer screening?**

**Yes No don’t know**

1. **Cervical cancer protection provided by HPV vaccine is:**

**100% 90% 70% 50%**

1. **Would you like to receive/advice HPV vaccination?**

**Yes No don’t know**

1. **What do you think will be the most important obstacle preventing yourself to receive/advice HPV vaccination?**

**High cost Worry about complications**

**Worry about efficacy of vaccine Inadequate information**

1. **What are your sources of knowledge and information on HPV vaccination?**

**Medical school teachings Friends News paper**

**Books Internet Television**

1. **Has anybody (friends/family) have sought your opinion till now regarding HPV vaccination?**

**Yes No**

1. **Would you like yourself to be educated by experts?**

**Yes No**

***Thank you for your participation***

***All information obtained in the study will be kept Confidential and used for medical research only***
